# Quantifying distinct associations on different temporal scales: comparison of DCCA and Pearson methods

**DOI:** 10.1038/srep36759

**Published:** 2016-11-09

**Authors:** Lin Piao, Zuntao Fu

**Affiliations:** 1 Lab for Climate and Ocean-Atmosphere Studies, Dept. of Atmospheric and Oceanic Sciences, School of Physics, Peking University, Beijing,100871, China

## Abstract

Cross-correlation between pairs of variables takes multi-time scale characteristic, and it can be totally different on different time scales (changing from positive correlation to negative one), e.g., the associations between mean air temperature and relative humidity over regions to the east of Taihang mountain in China. Therefore, how to correctly unveil these correlations on different time scales is really of great importance since we actually do not know if the correlation varies with scales in advance. Here, we compare two methods, i.e. Detrended Cross-Correlation Analysis (DCCA for short) and Pearson correlation, in quantifying scale-dependent correlations directly to raw observed records and artificially generated sequences with known cross-correlation features. Studies show that 1) DCCA related methods can indeed quantify scale-dependent correlations, but not Pearson method; 2) the correlation features from DCCA related methods are robust to contaminated noises, however, the results from Pearson method are sensitive to noise; 3) the scale-dependent correlation results from DCCA related methods are robust to the amplitude ratio between slow and fast components, while Pearson method may be sensitive to the amplitude ratio. All these features indicate that DCCA related methods take some advantages in correctly quantifying scale-dependent correlations, which results from different physical processes.

Identifying interesting relationships between pairs of variables is increasingly important for various disciplines, such as climatology, ecology, economy, neurology and so on. And in the course of quantifying relationships, what has caught our attention is the relationship between variables with multi-temporal scale features can be extremely different on different temporal scales^1^,^2^. For example, it has been found that precise neuronal synchronization often co-occurs with slow co-variation in neuronal rate responses^2^. When concentrating precise neuronal synchronization, a component with a 25 ms time scale should be investigated and positive correlation is presented in that process. While for co-variation, it is a slow process (100 ms time scale or larger) which displays negative correlation (see Fig. 1b in their paper^2^). The findings above aroused our thinking: with regard to meteorology, besides the associations are totally different between slow components or fast components, it is likely that both associations are of great concern because two kinds of components may correspond to different physical processes^3^. In this case, it is really necessary to find a proper method in revealing true distinct correlations on different timescales which corresponds to different physical processes. And only through better understanding different physical processes, meteorologists can improve their models’ physical parts and then improve weather foresting or climate prediction. So when coming to this situation will Pearson correlation still be useful for uncovering the true associations between slow components or fast components? This is the first question to be answered in this paper.

Besides the simplest Pearson correlation coefficient, many other correlation measures, such as rank correlation^4^, distance correlation^5^,^6^, Detrended Cross-Correlation Analysis (DCCA for short) cross-correlation coefficient^7^,^8^,^9^,^10^, have been proposed to quantify associations under specific situations. In this paper, we will restrict our analysis to the widely used DCCA cross-correlation coefficient, which is based on the detrended cross-correlations analysis^11^. The reason to choose DCCA related method lies in its original algorithm for calculating the correlation coefficient (see “Data and Method” section), where the restricted windows have been used. Most importantly, studies based on Monte Carlo simulations of ARFIMA(0, d, 0) processes indicate that the standard Pearson coefficient is practically *useless* for non-stationary time series and the DCCA coefficient is able to estimate the true correlation coefficient between series *precisely*^10^. In this case, DCCA cross-correlation coefficient may have ability to reveal true distinct associations on different temporal scales, which has not been reported in the previous literature.

Here comes another question: when dealing with meteorological data, whether extremely different correlations are really existed on different temporal scales? This question can be easily answered from an example of daily mean air temperature and relative humidity series, see Fig. 1, where the slow components of these two series are positively corrected (the Pearson coefficient is 0.61) but as indicated by the zoom-in, the fast components can be negatively corrected (the Pearson coefficient is −0.74). More detailed cross-correlations between these two fundamental meteorological variables for the period of 1955–2014 at four typical stations to the east of Taihang mountain in China are shown in Fig. 2, and the solid lines are for original data. The four stations are Beijing (39.48°N,116.28°E), Jinan (36.41°N,116.59°E), Shijiazhuang (38.02°N,114.25°E) and Zhengzhou (34.43°N,113.39°E), respectively. The black lines are the results from Pearson method, while the blue lines are the results from DCCA1 method (the local trend was calculated by using a linear trend fit). Here, we need to ***stress*** that we measure distinct associations on different temporal scales *directly* from the original series other than the filtered series or the successive differences of air temperature and relative humidity, which have been used in ref. 7, since the additional difference process will filter out most of the slow components of these two series. As we can see, on the whole, both the results from Pearson method and DCCA1 method have confirmed that the associations between mean temperature and relative humidity are distinct on different time scales: they are negatively correlated at fast variations, but positively correlated at slow variations. Here slow variations (around yearly time scale) of mean temperature and relative humidity are positively correlated, which shows the effect from the summer/winter monsoon process. When summer is coming, the temperature increases and the East Asian Summer Monsoon (EASM) system begins to influence east China. With EASM coming, a large amount of water vapor will be transported to east China which then can cause the relative humidity increases. The meteorological process is opposite when winter comes. What’s more, we find one apparent difference between two methods: when taking into account values, *ρ*_*DCCA*1_ is larger than *ρ*_*Person*_ about 0.35–0.4 on the annual time scale at all four stations. In this case, which method quantifies the true correlation at slow variations, the Pearson method or the DCCA1 method? By contrast, on the around monthly time scale the cross-correlation coefficient *ρ*_*DCCA*1_ is nearly of the same value (Beijing station: around −0.2, all the other stations: around-0.3) as *ρ*_*Person*_. The negative correlation may represent the Clausius-Clapeyron relation. When the mean temperature increases, for ideal gas, its saturation vapor pressure will increase correspondingly and if there’s no other vapor sources the relative humidity will decrease. But as we can see, the value of the negative correlation is relatively low for both methods. What’s the reason behind this? This is the second question to be answered in this paper.

The paper is organized as follows. In the “Results” section, through traditional statistics we first investigate the distinct associations of slow components and fast components between mean temperature and relative humidity and try to answer the two questions in last paragraph. Further we will illustrate some advantages of DCCA method in discovering distinct correlations on different temporal scales by conducting two idealized numerical tests. Then a brief conclusion and discussion are made in the following section. In the end of this paper, the data and the methods are described in details.

## Results

### Distinct correlations on different scales in meteorological data

In traditional statistical analysis, in order to attenuate the influence of the slower signal components on faster ones and vice versa and find correct cross-correlations on different temporal scales, one straightforward approach is simply using filter methods (including low-pass filter, high-pass filter, and band-pass filter) to reach target signals on different time scales^12^,^13^ and then calculate the Pearson correlation coefficient for the target signals.

Therefore, first we should find properly reasonable frequency, or band-width in carrying out filter method. See Fig. 3, at these four stations, the mean air temperature seem to be associated with relative humidity on annual time scale and around monthly time scale since the time-averaged wavelet spectrum analysis of the two variables have shown that they have two significant characteristic time scales of 1 year and around 1 month. Besides, we find that the relative humidity variable also has other significant characteristic time scales, such as 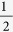 year or lager than 1 year, at the four stations. Since we only care about the consistency of the spectrum between mean air temperature and relative humidity, in the following part, through wavelet analysis, we try to explore the associations of the filtered out signals (annual cycle signal and 30 days high-pass filtered signal).

In Fig. 4, individual scatter plots of the zero mean processed annual cycle data at each station are shown. For all the four stations, the scatter plots displayed a roughly elliptical shape in the direction of bottom left to upper right, which indicates positive correlations between mean air temperature and relative humidity on annual time scale. The calculated Pearson correlation coefficients for the annual cycle data are 0.80 for Beijing station, 0.70 for Jinan station, 0.63 for Shijiazhuang station and 0.70 for Zhengzhou station. By comparing with the correlation coefficients for original data in Fig. 2, we can successfully answer the first question that although the correlations provided by DCCA1 method to the original data on annual time scale is a little bit smaller (around 0.05–0.15) than the values above, DCCA1 method is indeed still much better in providing true positive correlations between slow components when dealing with raw data, while Pearson method largely underestimates the positive correlations in this case. The reason behind this situation is, for DCCA1 method, the influence of fast components on slow components can be largely removed when larger restricted windows are employed, while it doesn’t work for Pearson method owing to both the fast components and the slow components remain to be sampled with their full variances. Further, what has drawn our attention is the elliptical shape in the scatter plots. For the purpose of illustrating what has resulted in the elliptical shape, we conducted an idealized numerical test. In Fig. 5a, there are three simulated cosinoidal signals, all having same frequency and same amplitude but different phases. The black and red lines have a phase lag of 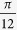, while the black and blue lines have a larger phase lag of 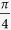. Figure 5b shows the scatter plots of these two simulated signals. The black one represents the perfect positive correlation between the black line and itself in Fig. 5a, while the red one with elliptical shape represents the positive correlation between the black line and the red line in Fig. 5a and the blue one is for the black line and the blue line in Fig. 5a. As we can see, the larger the phase lag between two signals, the larger the eccentricity is for the scatter plot with elliptical shape. Therefore the elliptical shapes in Fig. 4 may also result from the phase lag between mean air temperature and relative humidity (mean air temperature reaches extreme values first then relative humidity reaches extreme values). Moreover, the elliptical shape in Fig. 4d is much ambiguous than others which indicates that the phase lag between mean air temperature and relative humidity at Zhengzhou station is non-stationary. And for the other three stations, the phase lags are relatively stationary over time, and mean air temperature and relative humidity at Beijing station possesses the smallest phase lag, the second is at Jinan station and the last is at Shijiazhuang station.

**Figure 1:**
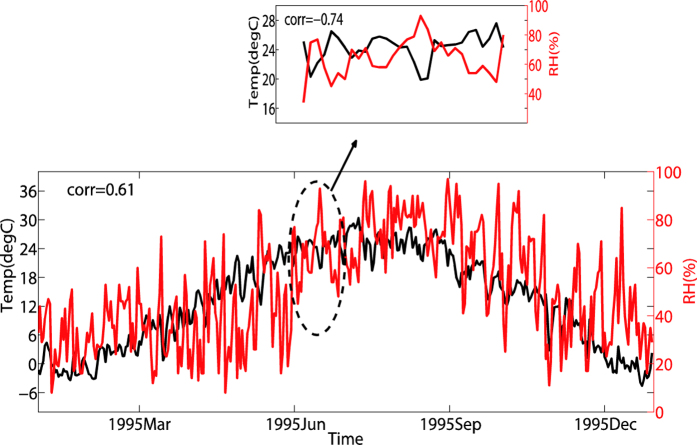
An example of restricted sampling by selecting only a sub-segment of daily mean air temperature and relative humidity series. The correlation is positive (0.61) for the entire (one year) segment and the correlation is negative (−0.74) for the sub-segment (only during June).

**Figure 2:**
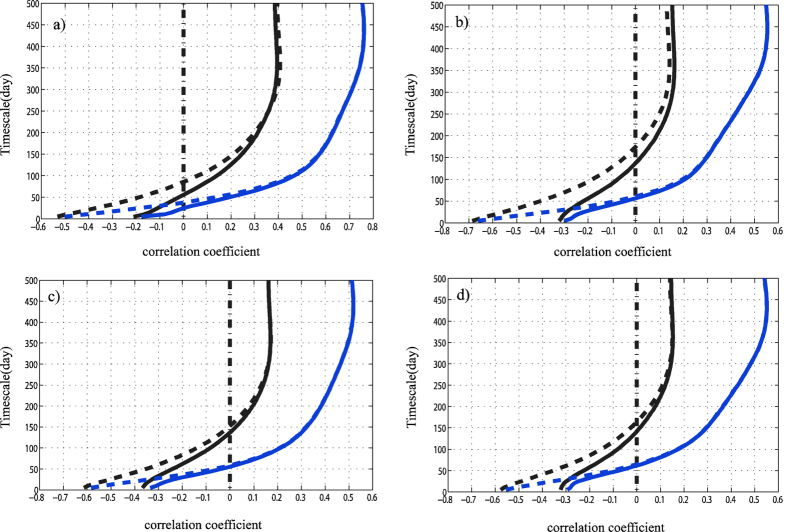
Correlations between mean temperature and relative humidity at different stations using Pearson (black line) and DCCA1 (blue line) method. The solid lines are for original data, while the dotted lines are for fast components in linked data. (**a–d**) are for Beijing, Jinan, Shijiazhuang and Zhengzhou station, respectively.

**Figure 3:**
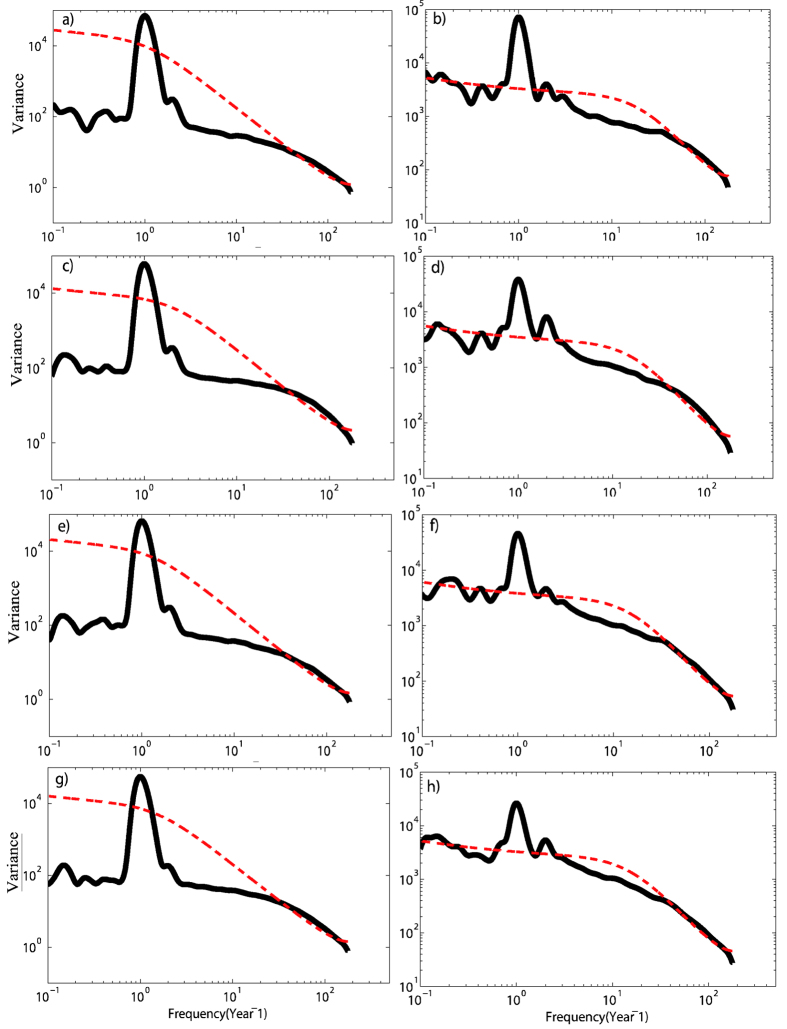
Time-averaged wavelet power spectrum of mean temperature and relative humidity at different stations (black lines). Left column is for the mean temperature and right column is for relative humidity. (**a,b**) are for Beijing station. (**c,d**) are for Jinan station. (**e,f**) are for Shijiazhuang station. (**g,h**) are for Zhengzhou station. The red lines are the 95% confidence power spectrum of the red-noise AR (1) process for each record.

**Figure 4:**
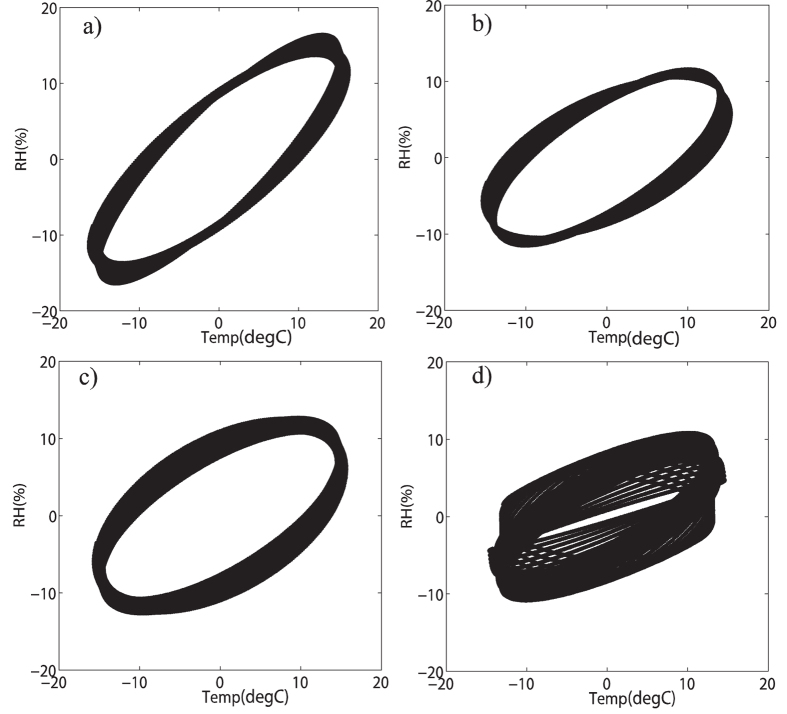
Scatter plots for zero-mean processed annual cycle data of mean temperature and relative humidity at different stations. (**a–d**) are for Beijing, Jinan, Shijiazhuang and Zhengzhou station, respectively.

**Figure 5:**
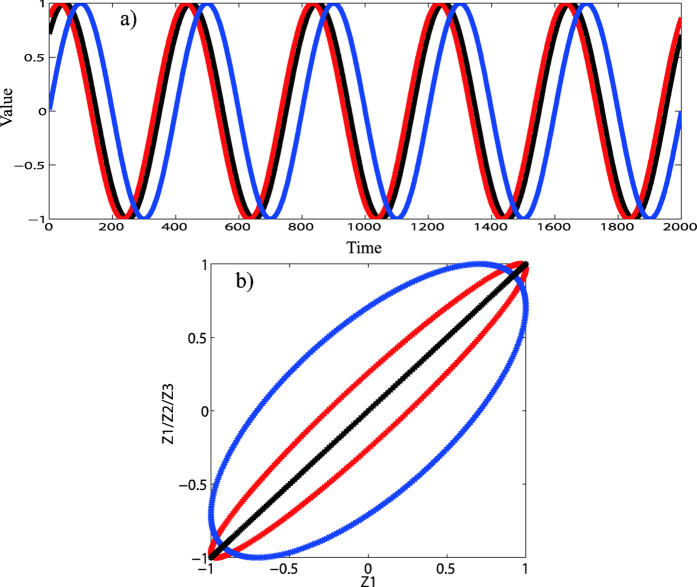
(**a**) Variations of three simulated cosinoidal signals, which have different phases. The black (referred as Z1) and red lines (referred as Z2) have a phase lag of π/2, while the black and blue lines (referred as Z3) have a larger phase lag: π/4 (**b**) Scatter plots for the simulated cosinoidal signals. The black one is for black line itself in (**a**). The red one is for the black and red lines in (**a**) and the blue one is for the black and blue lines in (**a**).

**Figure 6:**
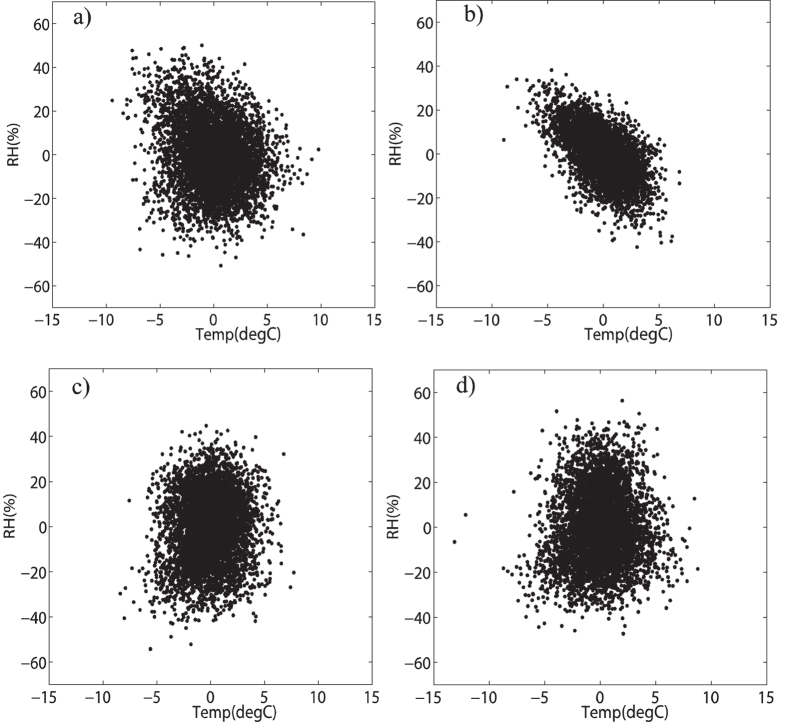
Scatter plots for zero-mean processed 30 days high-pass filter data of mean temperature and relative humidity at Beijing station during different seasons. (**a–d**) are for spring (MAM), summer (JJA), autumn (SON) and winter (DJF), respectively.

**Figure 7:**
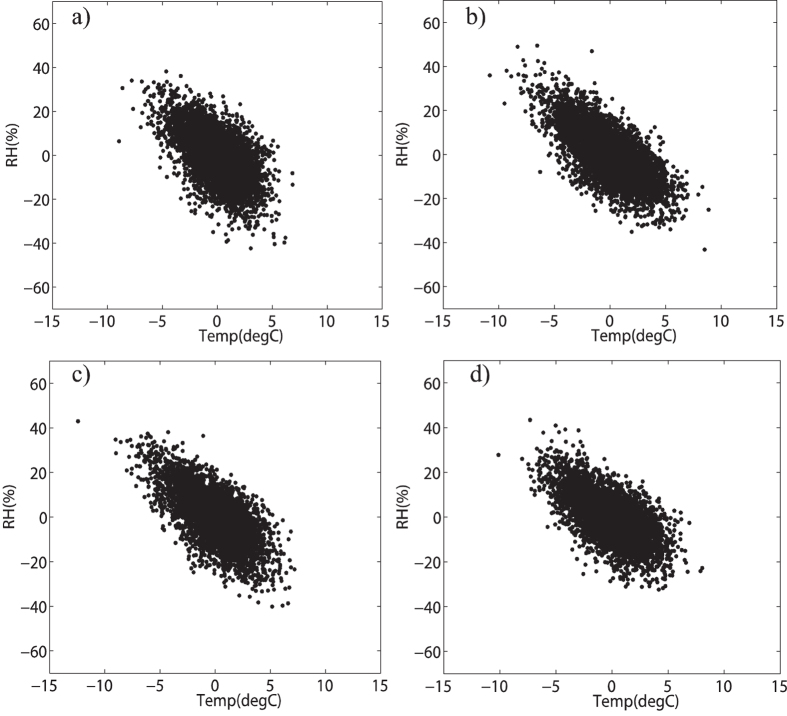
Scatter plots for zero-mean processed 30 days high-pass filter data of mean temperature and relative humidity at different stations during summer. (**a–d**) are for Beijing, Jinan, Shijiazhuang and Zhengzhou station, respectively.

Then Pearson correlation coefficients calculated for the 30 days high-pass filtered data are −0.15 for Beijing station, −0.30 for Jinan station, −0.38 for Shijiazhuang station and −0.32 for Zhengzhou station, which consists with the results from Pearson method and DCCA1 method to the unfiltered data in Fig. 2. The findings above indicate that both the Pearson method and DCCA1 method have nearly the same power in attenuating the correlations that emerge from the slow components and only retaining the correlations of fast components. This is achieved through restrictions in the sampling range of the raw data^2^. However the value of the negative Pearson correlation for the 30 days high-pass filtered data and the results in Fig. 2 from two methods are still relatively lower compared with the results showing in Fig. 1. And what’s the reason behind this? Taking Beijing station as an example, we examined the associations between mean air temperature and relative humidity over different seasons, see Fig. 6. The scatter plots show large differences among different seasons and the negative association is only much prominent for summer time (Fig. 6b). So does at the other stations. In this case, one possible reason for the low negative Pearson correlation to the 30 days high-pass filtered data and results from raw data in Fig. 2 at the four stations is that the Clausius-Clapeyron relation is non-stationary over time and it is only significant over summer. The correlations over other seasons attenuate the high negative correlation over summer, but they cannot fully cover up the high negative correlation over summer. Then the individual scatter plots for the zero mean processed 30 days high-pass filtered data at all the four stations are shown only during summer time, see Fig. 7. Significant negative correlations are shown at each station and the Pearson correlation coefficient at this time is −0.55 for Beijing station, −0.70 for Jinan station, −0.65 for Shijiazhuang station and −0.61 for Zhengzhou station. The absolute values of the negative correlation are largely improved (around 0.3–0.4). Unlike previous works (say, ref. 7), here we find the possible physical mechanism behind the distinct correlations on different timescales between air temperature and relative humidity. Moreover, we find the Clausius-Clapeyron relation is summer-preferred and is unsuitable in other seasons. If we hypothesize that the Clausius-Clapeyron relation is quite stationary through time on the time scale around one month, and in such situation does Pearson method or DCCA1 method can still provide us with the true correlations at fast variations? In order to accomplish this goal, we replaced the 30 days high-pass filtered data of other seasons with the summer time one and added the linked 30 days high-pass filtered data to the 30 days low-pass filtered data. Here we have leaved the slow component data unchanged and only made changes to the fast component data. The results of Pearson method and DCCA1 method for the new datasets are also shown in Fig. 2, see the dotted lines. The black dotted line is for the Pearson method, while the blue dotted line is for DCCA1 method. Both Pearson method and DCCA1 method can still retain the true correlations of fast components (below 30 days time scale). However, the Pearson correlation coefficient is not only altered dramatically at fast variations, it has also been changed a little bit at slow variations up to 300 days at Shijiazhuang station and Zhengzhou station and up to 500 days at Beijing station and Jinan station. While for the DCCA1 method, its value only changed dramatically on fast variations up to 50 days. The findings above indicate that Pearson correlation coefficient may be easily altered and it may not provide us with robust correlation estimations.

### Advantages of DCCA related method

We have demonstrated in the last part that DCCA method is quite robust in providing distinct correlations on different temporal scales, as we know that time series obtained from nature are always characterized with features that we may not fully understand and in this case we couldn’t fully understand the advantages of DCCA related method. Therefore in this part, in order to better illustrate the advantages of DCCA related method compared to Pearson method in offering true distinct correlation results on different temporal scales, we perform two idealized numerical tests using artificially generated continuous signals with known correlation characters which roughly corresponds to the cases for air temperature and relative humidity. The two generated slow components with a period of 365 days are perfectly positive correlated ( + 1), while the two generated fast components with a period of 30 days are perfectly negative correlated (−1). The detailed procedure in artificially generating signals is presented in the “Data and Methods” section. We systematically investigate the importance of and the dependencies between the following two factors: the signal to noise ratio (SNR) (investigated in Test I) and the ratio of the amplitudes (RA) between the slow and the fast components (investigated in Test II).

**Figure 8:**
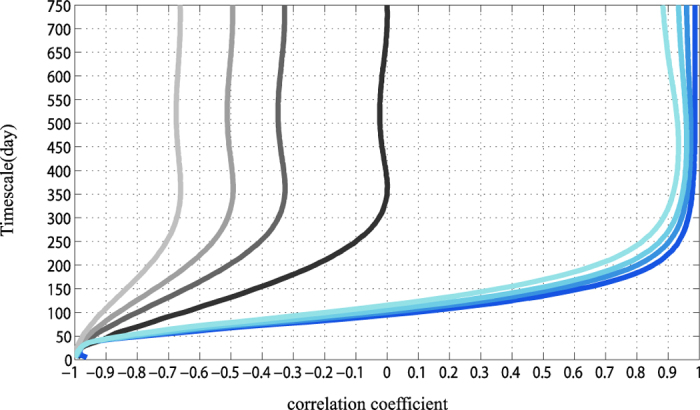
Correlations between simulated signals at different time scales for Test I: Constant RA of the slow and the fast components, while the SNR varies. The grey/blue lines represent the results of Pearson/DCCA1 Darkest lines are the results without adding noises. As the color of lines becoming lighter, the SNR we set up is smaller (SNR = 2.0/1.0/0.5).

Test I: At first, when no noises are added to the signal (see the darkest lines in Fig. 8). Both the DCCA1 method and the Pearson method have the ability in attenuating the perfect positive correlations from the slow components and maximally retaining the perfect negative correlation on the time scale of 30 days or lower. For Pearson method, it is achieved through restrictions in the sampling range of the original data. The restricted windows have helped in reducing the variance only for the slow components, whereas the fast components remain to be sampled with their full variances. As for the DCCA1 method, it is achieved most importantly through the detrending procedure which can largely remove the slow component as local linear trend. While for the time scale of 365 days or larger, it is impossible for Pearson method to unveil the true cross-correlation. And its value is close to 0 which indicates that the Pearson correlation coefficient reflects the superimposed influence of the slow components and the fast components. However, for the DCCA1 method, it can maximally weaken the negative correlations from the fast components and only remain the perfect positive correlation of the slow components. This is achieved by the calculating profile procedure which has increased the ratio of the variance between the slow components and the fast components.

Further, as we all know, all real-world signals are contaminated with measurement noise and we don’t want to get noisy relationships instead of signal relationships. Thus the negative correlated white noises are added to the signals and we want to test the robustness of DCCA1 and Pearson method in dealing with noises. See Fig. 8, the lighter the color of lines is, the smaller the SNR we set up is (SNR = 2.0/1.0/0.5). The smaller the SNR is, the stronger the noise is. As we can see, Pearson correlation coefficient is greatly influenced by noises. While for the DCCA1 cross-correlation coefficient, its value is only slightly influenced by the noise at slow variations. Even the SNR equals to 0.5, *ρ*_*DCCA*1_ is still around 0.93. Overall, we find that DCCA related method is quite superior over Pearson method in its insensitiveness to noises.

**Figure 9:**
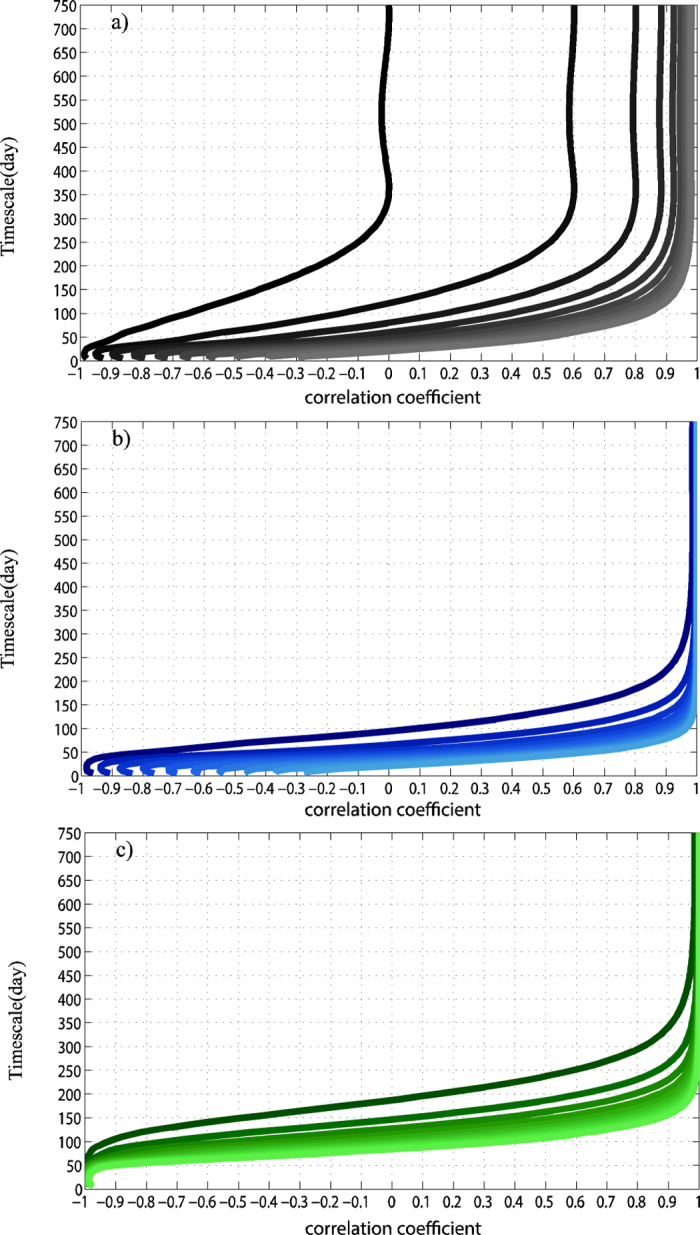
Correlations between simulated signals at different time scales for Test II:The SNR equals to 0, while the RA of the slow and the fast components varies. As the color of lines become lighter, the RA is larger (RA∈[1:10]). The grey (**a**), blue (**b**) and green (**c**) lines represent the results of Pearson/DCCA1/DCCA2.

Test II: Although for the case in our paper, the RA between the slow components and the fast components roughly equals to 1, it is likely in other cases that the RA is larger than 1. Therefore in this part we will test the sensitiveness of methods to the RA between the slow components and the fast components, see Fig. 9. The lighter the color of lines is, the larger the RA is. In Fig. 9a presents the results of Pearson correlation coefficient. As the RA becomes larger, Pearson method will be more able to discover the positive correlations on the time scale of 365 days or larger (close to +1) than the negative correlations on the time scale of 30 days or lower (far from −1) due to that the power of restricted windows has disappeared. As for the DCCA1 method, see Fig. 9b, as the RA becomes larger, it still has the full ability in uncovering the perfect positive correlations on the time scale of 365 days or larger. But unfortunately, it couldn’t reveal the perfect negative correlations on the time scale of 30 days or lower for larger RA, similar features can be found for Pearson method. When the RA equals to 1, the detrending procedure can largely remove the slow component as local linear trend. However, when the RA is larger, the slow components can not to be removed only as local linear trend. Higher order detrending must be taken into consideration. Actually if higher order detrending such as quadratic polynomial fit has been taken, both positive and negative correlations at the slow and fast variations can be fully uncovered by DCCA related methods. DCCA2 (the local trend was calculated by fitting the data using a polynomial fit of order 2) results are shown in Fig. 9c. Even as the RA becomes larger, DCCA2 method is still prove to be robust in providing both distinct correlations. To sum up, the relative RA strongly influences the performance of different methods.

In conclusion, the results found in idealized sequences with known correlation features indicate that DCCA related method is quite robust in providing a complete account of distinct correlations on different temporal scales. Under the assumption that Clausius-Clapeyron relation is stationary over time, the tests show that the Pearson correlation coefficient is easily to be altered and cannot provide us robust results. All these findings can be partially explained by the Test I and II.

## Conclusion and Discussions

In this paper, we have studied the phenomenon of distinct associations on different time scales and its quantification. We provided an example: the associations between daily mean air temperature and daily relative humidity at four stations over the regions to the east of Taihang mountain in China during the period of 1955–2014. In such case, they are negatively correlated around monthly time scale and positively correlated on annual time scale. The two time scales correspond to two different physical processes respectively which are equally important to meteorologists: fast variations present the Clausius-Clapeyron relation; slow variations are related to the monsoon process. Here we used two methods to quantify the associations: the simplest and most tractable Pearson correlation coefficient and the recently developed DCCA cross-correlation coefficient. On short time scale, both the Pearson method and DCCA1 method can properly reveal the true negative correlation. However, on the annual time scale Pearson method fails, while DCCA1 method has successfully provided the true results. Most importantly, DCCA1 method has been applied directly to the raw data and it is still proved to be useful in unveiling the distinct correlations on different time scales. While the Pearson method can only reach true correlations on different time scales by additional step: filtering the input signals prior to the computation of the cross-correlation on specific scale. And as we all know, filtering procedure can only be performed under the assumption of the principle of linear superposition, which is the case in our paper. However, when comes to boundary layer, the processes usually take nonlinear or chaotic features, and in such case, filtering procedure is not recommended since it can obscure the underlying deterministic structure of a chaotic process^14^. What’s more, in order to illustrate the advantages of DCCA related method over Pearson method in detecting distinct correlations on different time scales, we performed two idealized numerical tests. Test I shows DCCA1 not only has the ability in revealing the perfect negative correlations between fast components as Pearson does, it can also capture the perfect positive correlations between slow components that Pearson has failed to capture. Moreover, DCCA related method still can provide quite robust results even for slow components contaminated with noise. While Test II demonstrates the robustness of methods to the RA between slow and fast components. The RA of slow and fast components strongly influenced the performance of different methods. Pearson method may be not able to uncover distinct correlations on different time scales. However, DCCA related method have proved its robustness in providing the distinct correlations on different time scales. These findings in this paper indicate if such phenomenon also happens in other disciplines, the DCCA related method should be the first choice in uncovering it.

Furthermore, we need to note that in this study the numerical tests are only roughly corresponding with the real world cases for air temperature and relative humidity. There are many other factors we don’t take into consideration, such as quasi-periodic components on smaller scales, the varying correlations through time at smaller time scale and so on. Therefore, more detailed analysis is still required in future.

## Data and Methods

### Meteorological data

Observed daily air mean temperature and relative humidity data from 4 stations with a continuous record over 60-year (1955–2014) were provided by the China Meteorological Administration. The 4 stations are Beijing station (39.48°N,116.28°E), Jinan station (36.41°N,116.59°E), Shijiazhuang station (38.02°N,114.25°E) and Zhengzhou station (34.43°N,113.39°E), respectively, all are located to the east of Taihang Mountain in China.

### Simulated continuous signals

Based on the results for correlations between mean air temperature and relative humidity at different time scales, we find that the correlations between two variables may be totally different at different temporal scales: the slow components (at annual time scale) of two variables are positive-correlated, while the fast components (at monthly time scale) of two variables are negative-correlated. In order to illustrate whether DCCA related method can offer more correct correlation results than Pearson method and its advantages compared to Pearson method, in this part, we try to generate artificial continuous signals with known correlation characters roughly corresponding to the cases mentioned above for air temperature and relative humidity.

For Test I, we used combinations of cosinoidal components and high-frequency noise. We first generate two perfect cosinoidal signals, then constructed the first target signal as a sum of these two components and the second target signal as the slow component signal minus the fast signal, as follows


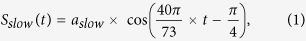



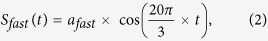



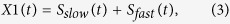



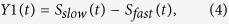


where *t* is time (*t*∈[0.01:80]) and sampling interval is 0.01, in this case, the data length we generated is 8000. Further we can see that 365 days and 30 days are the period of the slow and fast components and can be easily discriminated from each other. *a*_*slow*_ and *a*_*fast*_ are the amplitudes of the slow and fast component, respectively. For the Test I, we set both *a*_*slow*_ and *a*_*fast*_ equal to 1. Then the RA between the slow component and fast component also equal to 1. It is set to 1 because the final ratio roughly equals to 1 for mean temperature and relative humidity case. As we all know, all real-world signals are contaminated by measurement noise, in order to quantify the robustness of each method in dealing with noise, we also generated other target signals by mixing *X*1(*t*) and *Y*1(*t*) with noise. For *X*1(*t*) we added the noise to it, while for *Y*1(*t*) we subtracted the same noise from it, which indicates negatively correlated noise is added to the signals. The noise we used is the simplest and most tractable white noise: sequences of independently identically distributed random numbers. Three different level of noise is added using the measurement of signal-to-noise ratio (SNR), SNR = 2.0, SNR = 1.0 and SNR = 0.5, respectively.

For Test II, we investigate the sensitiveness of the quantifying methods to the RA of the slow and the fast components. For this situation, we set *a*_*fast*_ equal to 1 and vary *a*_*slow*_ from 1 to 10, which may correspond to other cases that the slow components always have larger amplitude than the fast components.

### Methods

In this subsection, we briefly explain how to calculate DCCA cross-correlation coefficient briefly.

Consider two time series 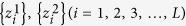. First, we calculate the random walk profile by integrating the series, using


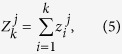


where *j* = 1, 2, *k* = 1, 2, 3, …, *L*. Second, we divide each profile into *L*−*s* overlapping windows, and each window contains *s* + 1 values. For the profiles, in each window that begins at *i* and ends at *i* + *s*, we define the local trend 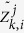
(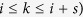 using a *q*-order polynomial fit. Then, we acquire the detrended walk as the difference between the original walk and local trend.

Next we calculate the covariance of the residuals in each window:


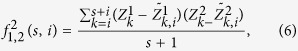


Finally, we calculate the detrended covariance by summing over all overlapping *L*−*s* windows of the scale *s*, we get:


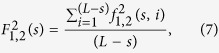


In the next step, we calculate the DCCA cross-correlation coefficient^9^, which is defined as the ratio between the detrended covariance function 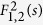 and the detrended variance function 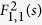 and 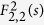:


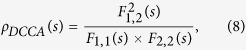


where *ρ*_*DCCA*_(*s*) is the DCCA cross-correlation coefficient on time scale of *s*. The value of *ρ*_*DCCA*_(*s*) has a range of [−1, 1].

The Pearson correlation coefficient on the time scale of *s* is also calculated in a similar way, but to the original data 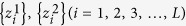 which also have been divided into *L*−*s* overlapping windows, and each window contains *s* + 1 values.

Moreover, the statistical technique used in this study is wavelet analysis^15^,^16^,^17^.

## Additional Information

**How to cite this article**: Piao, L. and Fu, Z. Quantifying distinct associations on different temporal scales: comparison of DCCA and Pearson methods. *Sci. Rep.* **6**, 36759; doi: 10.1038/srep36759 (2016).

**Publisher’s note:** Springer Nature remains neutral with regard to jurisdictional claims in published maps and institutional affiliations.
